# Causal Effect of Donor Source on Survival of Renal Transplantation Using Marginal Structural Models

**Published:** 2018-05

**Authors:** Amir ALMASI-HASHIANI, Mohammad Ali MANSOURNIA, Abdolreza REZAEIFARD, Kazem MOHAMMAD

**Affiliations:** 1. Dept. of Epidemiology and Reproductive Health, Reproductive Epidemiology Research Center, Royan Institute for Reproductive Biomedicine, ACECR, Tehran, Iran; 2. Dept. of Epidemiology and Biostatistics, School of Public Health, Tehran University of Medical Sciences, Tehran, Iran; 3. Dept. of Epidemiology, School of Health, Shiraz University of Medical Sciences, Shiraz, Iran

**Keywords:** Cox regression model, Fractional polynomials, Inverse probability weighting, Marginal structural model, Renal transplantation, Stabilized weight

## Abstract

**Background:**

Marginal Structural Models (MSMs) are novel methods to estimate causal effect in epidemiology by using Inverse Probability of Treatment Weighting (IPTW) and Stabilized Weight to reduce confounding effects. This study aimed to estimate causal effect of donor source on renal transplantation survival.

**Methods:**

In this cohort study, 1354 transplanted patients with a median 42.55 months follow-up in Namazee Hospital Transplantation Center, Shiraz from Mar 1999 to Mar 2009, were included to use marginal structural Cox regression, binomial logistic regression model to estimate causal effect of donor source on the survival of renal transplantation. IPTW and stabilized inverse probability of treatment weighting are used as weights.

**Results:**

The un-weighted (crude) hazard ratios for live unrelated donor and deceased donor in comparison to live related donor as reference group was (HR: 1.03, 95% CI: 0.58–1.83, *P*=0.89) and (HR: 2.69, 95% CI: 1.67–4.31, *P*=0.001), respectively. Using a marginal structural Cox regression model and by stabilized weight, the hazard ratios for live-unrelated donor and cadaveric donor were (HR: 1.08, 95% CI: 0.47–2.45, *P*=0.84) and (HR: 3.63, 95% CI: 1.59–8.26, *P*=0.002), respectively. There was no difference between estimated effect size from marginal structural Cox regression, marginal structural logistic regression, and marginal structural Weibull regression model.

**Conclusion:**

There is no difference between related and unrelated donor source hazard ratio; however, hazard ratio for cadaveric donor was 3.63 times of hazard ratio for related donor and 3.34 times of it for unrelated donor. Therefore, the live donor (related or unrelated) has a better survival of renal transplantation than cadaveric donor.

## Introduction

Renal transplantation is the best therapy for end-stage renal disease (ESRD) and in comparison to peritoneal dialysis and hemodialysis is of a better long-term survival and better quality of life that are also cost-effective for public health (1–5). The limited availability of eligible and appropriate donor kidneys is the main limitation of renal replacement therapy in end-stage renal diseases (6).

Grafts and patients survival rates are favorable for those who receive kidneys from living kidney donors (related or unrelated) (5) but due to shortage in living kidney donors, cadaveric donors is a favorable alternative approach. Many factors affect the graft and patients survival rate such as donor’s variables, recipient’s variables and some factors related to operation procedure. Using traditional statistical methods, one of the main factors affecting the survival rate is type of renal donor (related, unrelated or cadaveric) (7, 8). There are several important variables confounding the causal effect of donor source on graft survival. To overcome this problem, one can apply causal methods which provide marginal causal effect estimates under certain assumptions (9–11). Marginal structural models (MSMs) are a new class of causal models in which the parameters can be consistently estimated by inverse probability of exposure weighting (12–18) as described for the first time (9, 19).

The objectives of this research were to determine the causal effect of donor source on graft survival rate using stabilized inverse probability weighted estimators of marginal structural models.

## Methods

### Participants

Study design has been reported in details in previously published papers (3, 4). This retrospective cohort study was aimed to consider the marginal effect of donor source on graft survival in 1356 kidney transplantation cases in Namazee Hospital Transplantation Center, Shiraz, Southern Iran, from Mar 1999 to Mar 2009. The exact time of transplantation was considered as “initial event” and “end-point event” was defined as the time when renal allograft was diagnosed to be absolutely and irreversibly non-functioning due to any cause including rejection and patient requirements of ordinary dialysis again. Cases were censored at the time of their last follow-up, which is, death due to events other than renal transplantation or Mar 2009 (end of study), whichever came first.

Overall, 1354 transplanted patients in this 10-yr period (with 60650.8 person months of follow up) were included in the study. Of those, 68 individuals were excluded due to lack of follow-up; remained participants were followed until Mar 2009 with a median 42.55 months follow-up.

### Potential confounders

Potential confounder variables were donor’s and recipient’s age, donor’s and recipient’s gender (male/female and same or different), donor’s and recipient’s blood groups (ABO and same or different), recipient’s immunosuppressive drug regimen, recipient’s marital status, underlying cause of ESRD, recipient’s weight, creatinine level at discharge and the duration of dialysis therapy before transplantation.

### Exposure

The exposure of interest was donor source of graft. Donor source was categorized into three groups of; live related donor, live unrelated donor and cadaveric donor (or deceased donor).

### Data collection

All required data were collected through reviewing of patients’ hospital records. The organ survival and every patient’s needs to regular dialysis were assessed and determined by nephrologists, follow-up clinics, and related institutions such as “Management Center for Transplantation and Special Diseases” and “Renal Patients Support Society”.

### Statistical methods

A marginal structural Cox regression model was used to measure the effect of donor source on graft survival rate quantified by HR (95% CI). A polytomous logistic regression model was used to estimate the probabilities of different categories of donor source. The reciprocal value of these probabilities, stabilized by multiplying the marginal probability of the exposure, they was used as stabilized weights in an unadjusted Cox regression analysis (16–18, 20–22).

In the modeling of exposure (donor source), fractional polynomials of Royston (23) were used, to discover the proper scale of continuous variables (i.e. donor’s and recipient’s age, recipient’s weight, creatinine level at discharge and the duration of dialysis therapy before transplantation).

Proportionality of hazard that is the stability of effect of a covariate over time, is one of the most important assumptions in Cox regression model (24). This assumption was graphically tested by two graphs. The graph of -log-log (S(t)) curves for levels of donor source against log (t) and the graph of observed (Kaplan-Meier curve) versus predicted values. Stata software, ver. 13 (Stata Corp, College Station, TX, USA) was used for all statistical analyses. The *P*-value of less than 0.05 was considered to be statistically significant.

## Results

### Characteristics of the sample

The results obtained from the preliminary analysis of baseline characteristics are presented in Table 1. The mean (SD) age of donors and recipients were 31.01 (11.21) and 34.99 (13.95) yr, respectively. 65.83% of donors and 64.35% of recipients were male. The donor and recipient sex in the 53.04% of cases were same and in 64.96% were different. In 84.96% of the cases, donor and recipient blood groups were same. In terms of marital status, 75.12% of donors and 70.73% of recipients were married.

**Table 1:** The frequency statistics of baseline characteristic of patients based on graft status (n=1354)

***Variables***		***Censored***	***Rejected***
Donor’s gender	Male	769 (91.11)	75 (8.89)
	Female	409 (92.74)	32 (7.26)
Recipient’s gender	Male	765 (91.73)	69 (8.27)
	Female	414 (91.80)	37 (8.20)
Gender composition	Same	620 (90.51)	65 (9.49)
	Different	557 (93.14)	41 (6.86)
Donor’s blood group	A	287 (89.97)	32 (10.03)
	B	237 (89.10)	29 (10.90)
	AB	52 (94.55)	3 (5.45)
	O	599 (93.3)	43 (6.70)
Recipient’s blood group	A	312 (90.17)	34 (9.83)
	B	275 (89.87)	31 (10.13)
	AB	73 (94.81)	4 (5.19)
	O	518 (93.33)	37 (6.67)
Blood group composition	Same	993 (91.35)	94 (8.65)
	Different	181 (93.78)	12 (6.22)
Donor source	Live related	362 (93.54)	25 (6.46)
	Live unrelated	391 (94.44)	23 (5.56)
	Deceased	427 (87.86)	59 (12.14)
Donor’s age	Mean (S.D)	30.76 (10.96)	33.92 (13.77)
Recipient’s age	Mean (S.D)	35.17 (13.85)	31.19 (14.48)

The mean (SD) weight of donors and recipients were 65.31 (11.98) and 57.73 (15.02) kg, respectively.

Overall, 403 cases (29.76%) were live related donor, 440 cases (32.5%) were live unrelated donor, and 511 cases (37.74%) were deceased donor. In 53.91% of patients, cause of end-stage renal disease (ESRD) was not known, but among cases with known cause, glomerulonephritis was more frequent (12.78%).

The rate of graft rejection was 1.76 (CI 95%: 1.46–2.13) cases per 1000 person-month follow-up. The rate for live related donor, live unrelated donor, and for deceased donor was 1.09 (CI 95%: 0.73–1.61), 1.17 (CI 95%:0.78–1.76), and 3.24 (CI 95%: 2.51–4.18) cases per 1000 person-month follow up respectively.

### Effect of donor source

**Fig. 1:**
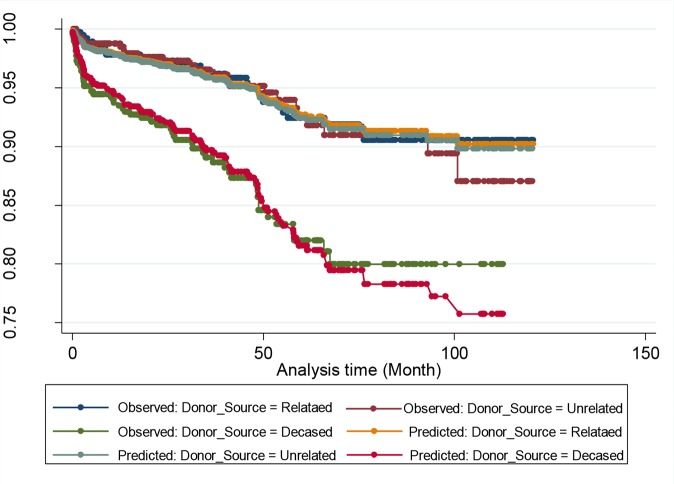
Graph of observed versus predicted values for assessing PH assumption

**Fig. 2:**
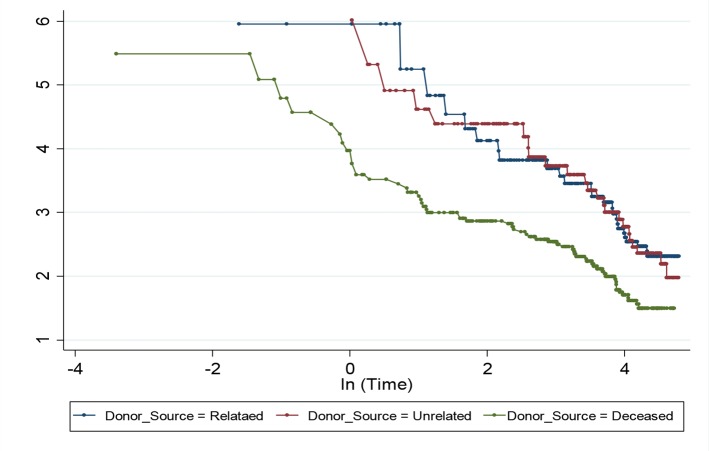
Graph of -log-log(S(t)) curves for levels of donor source against log(t) for assessing PH assumption

PH assumption for Cox regression model was tested for donor source. The stabilized inverse probability weights had a mean of 1.01 (SD= 2.26); the mean is about 1 as it should be, and SD is greater than the mean because the distribution of the weights was skewed to the right. In a sensitivity analysis, we truncated the weights at the first and 99th percentiles, but truncation does not change substantially the results (Fig. 1 and 2).

Interestingly, the unadjusted hazard ratio for renal transplantation among unrelated donors and deceased donor compared to the related donor was 1.03 (95% CI: 0.58, 1.83, *P*=0.89), and 2.69 (95% CI: 1.67–4.31, *P*=0.001) respectively (Table 2). The adjusted hazard ratio obtained from multiple Cox regression model for unrelated donor and deceased donor was 1.78 (95% CI: 0.79, 3.99, *P*=0.16) and 3.72 (95% CI: 1.78–7.78, *P*=0.001) respectively. The inverse probability-of-exposure-weighted hazard ratio from marginal Cox regression model for unrelated donor and in deceased donor was 1.08 (95% CI: 0.47, 2.54, *P*=0.84) and 3.63 (95% CI: 1.59–8.26, *P*=0.002) respectively. Hazard of graft rejection in unrelated and deceased donor is 1.08 and 3.63 times more than related donor.

**Table 2:** Hazard ratios between donor source and the survival of renal transplantation using different regression models

***Model type***	***Donor source***	***HR***	***95% CI***		***P***
			***Lower***	***Upper***	
Simple (unadjusted) Cox regression model	Related	1	-	-	-
	Unrelated	1.03	0.58	1.83	0.89
	Deceased	2.69	1.67	4.31	0.001
Standard multivariable Cox regression model	Related	1	-	-	-
	Unrelated	1.78	0.79	3.99	0.16
	Deceased	3.72	1.78	7.78	0.001
Weighted Cox regression model using stabilized weights	Related	1	-	-	-
	Unrelated	1.08	0.47	2.54	0.84
	Deceased	3.62	1.59	8.24	0.002

Compared to the unrelated donor, the inverse probability-of-exposure-weighted hazard ratio in deceased donor was 3.35 (95% CI: 1.46–7.68, *P*=0.004).

## Discussion

No data was found on the causal effect of donor source on the survival of renal transplantation. By means of a marginal structural model, the results of this study indicate that graft survival for unrelated donor was comparable with related donor but hazard of graft rejection for deceased donor was higher than that of related donor. Moreover, hazard ratio in deceased donor was 3.35 higher than the ratio for unrelated donor. Totally speaking, the live donor has an improved graft survival compared to deceased donor in renal transplantation.

A necessary requirement for correct exposure model specification is that the stabilized weights should have a mean of one (25, 26). If the stabilized weight means is not close to one, this can indicate a violation of some of the model assumptions or a misspecification of the weight models. In this study, the mean of stabilized weights was 1.01.

Because of some progress in transplantation process (such as patient care and enhanced immunosuppressive protocols), the outcome of renal transplantation has improved in recent years. The outcome has improved for both living (related and unrelated) donor and deceased donor recipients, but as our findings showed, living donor recipients have a better graft survival rate (27). A better selection of donors (such as a better genetic match), immediate surgical operation, opportunity to schedule operation electively when donor and recipient are in the best situation, lack of brain death and short ischemic time are the main reasons of better outcome for living donor transplants (28).

Our results showed that deceased donor has the worst graft survival rate. Cold ischemia time play an important role in the graft survival rate for deceased donor renal transplantation. “The association between extended cold ischemia time and inferior renal allograft outcomes are shown in deceased donor transplantation” (29). A high rate of delayed graft function, reduced graft survival and acute rejection in deceased renal transplants has been associated with lengthened cold ischemic time (30).

In a study among more than 150000 recipients transplanted from 1987 to 1997 at transplant centers, the best renal graft survival rate could be found among living donors whereas the worst survival rate could be found among cadaver donors (31). This difference was due to better genetic matching among living donors (especially in related donors) and also higher HLA compatibility between the donor and the recipient among living donors.

The marginal structural model estimates the net effect of donor source on graft survival rate. According to our findings, marginal structural model is better than other adjustment methods such as stratification; nevertheless, this model has restrictions, such as the assumption of no unmeasured confounders (32).

The main finding of this paper is that the marginal hazard ratio for unrelated donor was not significantly different than the ratio for related donors, but hazard ratio in deceased donor was 3.63 times higher than of ratio for related donors. In addition, the marginal hazard ratio for deceased donors was 3.35 higher than the ratio for unrelated donors. The live donors (related or unrelated) have a better survival of renal transplantation than deceased donors.

As one of the main limitations of the study, we conducted a retrospective cohort study and in few cases we used registered phone number to ascertain the outcome. Large prospective cohort studies are recommended to confirm the results of our study.

## Conclusion

The live donor (related or unrelated) has a better survival of renal transplantation than cadaveric donor. There is no difference between related and unrelated donor source hazard; but hazard for cadaveric donor was 3.63 times of hazard for related donor and 3.34 times of it for unrelated donor.

## Ethical considerations

Ethical issues (Including plagiarism, informed consent, misconduct, data fabrication and/or falsification, double publication and/or submission, redundancy, etc.) have been completely observed by the authors.
